# Supplementary material for: Circulating microRNAs as Early Biomarkers of Colon Cancer: A Nested Case-Control Study Within a Prospective Cohort
Source: Int J Mol Sci. 2025 Aug 15;26(16):7893. doi: 10.3390/ijms26167893 (PMC12386545; doi:10.3390/ijms26167893)
Supplement: Supplementary file 1 [file ijms-26-07893-s001.zip › ijms-3778590-supplementary.pdf]

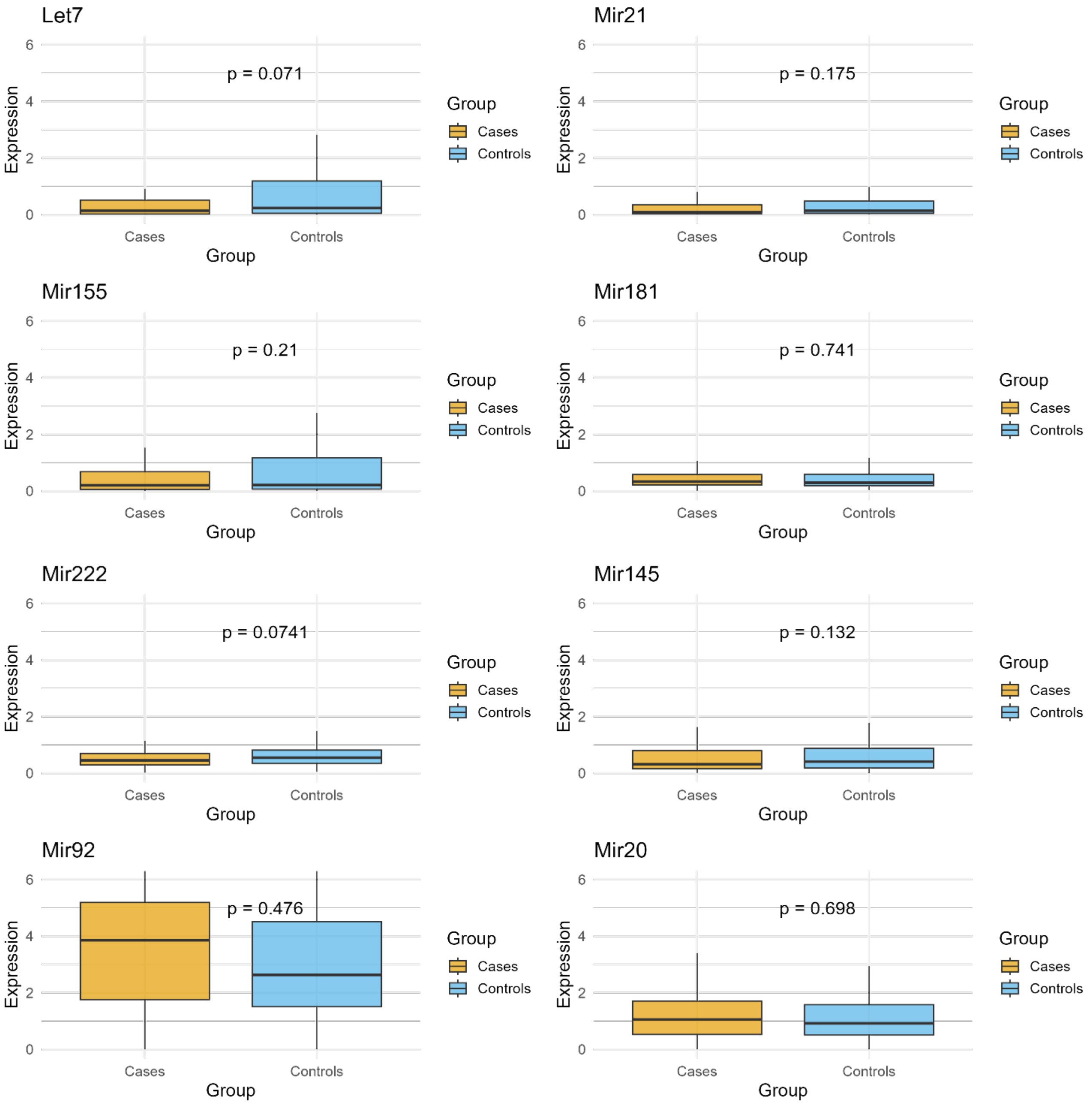

Supplementary Figure S1. Expression levels of eight selected miRNAs (Let7, Mir21, Mir155, Mir181, Mir222, Mir145, Mir20, Mir92) normalized to Mir484 in incident colon cancer cases and healthy controls. Boxplots show the median (horizontal line) with interquartile range (box). P-values from Wilcoxon rank-sum tests comparing expression between groups are annotated on each plot.
